# Radiologist agreement on the quantification of bronchiectasis by high-resolution computed tomography

**DOI:** 10.1590/0100-3984.2015.0146

**Published:** 2017

**Authors:** Milene Carneiro Barbosa de Brito, Maurício Kenji Ota, Fernando Sergio Studart Leitão Filho, Gustavo de Souza Portes Meirelles

**Affiliations:** 1 MD, MSc, Radiologist at the Clínica da Imagem do Tocantins, Araguaína, TO, Brazil.; 2 MD, Radiologist for the Fundação Instituto de Pesquisa e Estudos de Diagnóstico por Imagem (FIDI), São Paulo, SP, Brazil.; 3 PhD, Professor of Medicine at the Universidade de Fortaleza (Unifor), Fortaleza, CE, Brazil.; 4 PhD, Coordinator of the Thoracic Imaging Team of the Grupo Fleury, São Paulo, SP, Professor and Advisor for the Graduate Course in Clinical Radiology at the Escola Paulista de Medicina da Universidade Federal de São Paulo (EPM-Unifesp), São Paulo, SP, Brazil.

**Keywords:** Bronchiectasis, Lung, Tomography, X-ray computed, Observer variation

## Abstract

**Objective:**

To evaluate radiologist agreement on the quantification of bronchiectasis by high-resolution computed tomography (HRCT).

**Materials and Methods:**

The HRCT scans of 43 patients with bronchiectasis were analyzed by two radiologists, who used a scoring system to grade the findings. Kappa (κ) values and overall agreement were calculated.

**Results:**

For the measurement and appearance of bronchiectasis, the interobserver agreement was moderate (κ = 0.45 and κ = 0.43, respectively), as was the intraobserver agreement (κ = 0.54 and κ = 0.47, respectively). Agreement on the presence of mucous plugging was fair, for central distribution (overall interobserver agreement of 68.3% and κ = 0.39 for intraobserver agreement) and for peripheral distribution (κ = 0.34 and κ = 0.35 for interobserver and intraobserver agreement, respectively). The agreement was also fair for peribronchial thickening (κ = 0.21 and κ = 0.30 for interobserver and intraobserver agreement, respectively). There was fair interobserver and intraobserver agreement on the detection of opacities (κ = 0.39 and 71.9%, respectively), ground-glass attenuation (64.3% and κ = 0.24, respectively), and cysts/bullae (κ = 0.47 and κ = 0.44, respectively). Qualitative analysis of the HRCT findings of bronchiectasis and the resulting individual patient scores showed that there was an excellent correlation between the observers (intraclass correlation coefficient of 0.85 and 0.81 for interobserver and intraobserver agreement, respectively).

**Conclusion:**

In the interpretation of HRCT findings of bronchiectasis, radiologist agreement appears to be fair. In our final analysis of the findings using the proposed score, we observed excellent interobserver and intraobserver agreement.

## Abstract (pt)

**Objetivo:**

Avaliar a concordância entre radiologistas na quantificação de bronquiectasias pela tomografia computadorizada de alta resolução (TCAR).

**Materiais e Métodos:**

Foram avaliadas TCARs de 43 pacientes com bronquiectasias. Os achados foram graduados segundo um escore, e os valores de kappa (κ) e concordância geral foram calculados.

**Resultados:**

Para o tamanho e a aparência das bronquiectasias, houve concordância moderada interobservador (κ = 0,45 e κ = 0,43, respectivamente) e intraobservador (κ = 0,54 e κ = 0,47, respectivamente). Para a presença de impactação mucoide, a concordância foi mediana (central: concordância geral de 68,3% interobservador e κ = 0,39 intraobservador; periférica: κ = 0,34 interobservador e κ = 0,35 intraobservador). Para espessamento brônquico, a concordância foi mediana (κ = 0,21 interobservador e κ = 0,30 intraobservador). Na análise de opacidades, vidro fosco e cistos/bolhas, a concordância foi mediana (κ = 0,39, concordância geral de 64,3% e κ = 0,47 interobservador e concordância geral de 71,9%, κ = 0,24 e κ = 0,44 intraobservador, respectivamente). Após análise qualitativa dos achados de bronquiectasias na TCAR, realizou-se um escore final para cada paciente, com excelente correlação entre os observadores (coeficiente de correlação intraclasse de 0,85 interobservador e 0,81 intraobservador).

**Conclusão:**

Os achados tomográficos de bronquiectasias apresentaram concordância regular entre os observadores. Na análise final dos achados, foi encontrada excelente correlação entre os observadores e na avaliação do mesmo observador.

## INTRODUCTION

Irreversible bronchial dilatation, whether localized or diffuse. is known as bronchiectasis. Typically, it is the result of chronic infection, obstruction of nearby airways, or congenital bronchial abnormalities that lead to infection, such as cystic fibrosis or ciliary dyskinesia^(1)^. Bronchiectasis affects between 1/1,000 and 1/5,000 people in the general population^(2)^.

When there is clinical suspicion of bronchiectasis, the investigatory algorithm uses imaging methods for diagnosis, including simple thoracic radiography and high-resolution computed tomography (HRCT) of the lungs. HRCT is currently considered the method of choice for diagnosis. The protocol of acquiring images with slices of 1.0-1.5 mm in thickness, at 10 mm intervals, shows a sensitivity and specificity of 98% and 93-99%, respectively^(3-5)^.

The use of quantifying systems for bronchiectasis, using imaging examinations—also known as radiological scores—allows abnormal findings to be standardized and correlates well with functional test results and with quality of life^(6-8)^, such systems being used as tools for clinical monitoring of patients and their therapeutic response^(9)^.

The first bronchiectasis quantification system devised for simple radiography was presented in 1958^(10)^. Since then, a series of quantification systems using HRCT have been developed^(11-18)^, and others have recently been proposed for tomosynthesis^(19)^ and magnetic resonance imaging^(20)^. However, the majority of those systems are based on tomography findings in patients with cystic fibrosis.

To our knowledge, there have been no studies using HRCT to quantify bronchiectasis among adults in developing nations such as Brazil. The objective of the present study was to evaluate radiologist agreement for the quantification of bronchiectasis using HRCT scans and for the identification of the findings associated with the disease, such as mucous plugging, bronchial wall thickening, and pulmonary involvement, using the scoring system proposed by Brody et al.^(18)^.

## MATERIALS AND METHODS

This was a prospective observational study. We selected 43 HRCT scans of adult patients diagnosed with bronchiectasis at the pulmonology outpatient clinic of a tertiary reference center between March and June of 2008. In all of the patients, the diagnosis of bronchiectasis was based on clinical data and imaging findings. The patients were included consecutively at the time of their outpatient visit. The study was approved by the research ethics committee of the institution, and all participating patients gave written informed consent.

During the clinical follow-up, the patients underwent HRCT scans of the chest, as requested by the attending physician. The tomography slices were obtained according to the following criteria: in the supine position; without intravenous contrast; 1-2 mm in thickness; an increase of 10 mm; a high-resolution filter; a matrix of 512 × 512; and an exposure time of 1-2 s per slice. The images obtained were documented with windows appropriate for the study of the lung parenchyma (window width: 1000 to 1500; window level: -600 to -800) and of the mediastinum (window width: 300 to 400; window level: 50 to 80). The expiratory phase was carried out in 18 patients, but in only 10 of these was it considered viable for analysis by radiologists. Cases in which there was convexity of the posterior wall and no significant reduction of the tracheal lumen during the expiratory phase were excluded, as were those in which there were respiratory movement artifacts that hampered the correct analysis of the images.

The HRCT scan images were analyzed by two radiologists, both with less than five years' experience in thoracic radiology, who had received standard training for HRCT during their academic studies and prior guidance regarding the evaluation and classification of examinations from another radiologist, with more than five years' experience in thoracic imaging. The readings were carried out in an independent and blind manner. The two evaluating radiologists were blinded to the patient history and clinical data, which could influence the evaluation of the imaging findings. They evaluated the examinations on tomography films, on a light box. One of the radiologists carried out a subsequent analysis of the HRCT scans 30-90 days after, with the aim of evaluating intraobserver agreement.

Using the Brody et al. score^(18)^, each lung lobe (considering the lingular segment of the right upper lobe to be a separate lobe) was evaluated separately in terms of the presence, size, and extension of the bronchiectasis, as well as the related findings, such as mucous plugging, bronchial wall thickening, parenchymal opacities, areas of ground-glass attenuation, cysts/bullae, and air trapping.

The final score was calculated by summing the points of each specific finding of all six lobes, multiplied by the severity of the involvement. We standardized the scores on a scale that ranged from 0 to 100, higher values indicating greater disease severity.

Subsequently, the interobserver and intraobserver agreement were evaluated for the quantification of the bronchiectasis and its associated findings on the HRCT scans. For the analysis of agreement, we used the kappa statistic (κ), with 95% confidence intervals, which is useful for the categorization of the variability obtained by the interpretation of the two data sets. For the categorization of bronchiectasis, agreement was classified as follows: κ < 0.00. no agreement; κ of 0.00-0.20, slight; κ of 0.21-0.40, fair; κ of 0.41-0.60, moderate; κ of 0.61-0.80, substantial; κ of 0.81 to 1,00, near perfect^(21)^.

In cases in which we did not analyze all of the categories in either of the evaluations, the κ test could not be applied. On those occasions, the calculation of general agreement was used: higher values indicating greater closeness between the evaluations of the observers.

The interobserver agreement for the final bronchiectasis score of each patient was determined in two ways. The interclass correlation coefficient shows the level of agreement and reproducibility between the two evaluations. Conover^(22)^ established the following criteria for the interclass correlation coefficient: ≥ 0.75, excellent correlation; ≥ 0.40 and < 0.75, moderate correlation; < 0.40, weak correlation.

A Bland-Altman graph^(23)^ determines the agreement between two measuring methods, representing the difference between each pair of values by their means. To determine the limit of agreement on the graphs, the standard deviation of the mean of the difference was considered twice.

## RESULTS

We evaluated the HRCT scans of 43 patients (24 women and 19 men). Ages ranged from 15 to 78 years (mean, 46.5 years). The causative factors of bronchiectasis most often identified were repeat infections and tuberculosis sequelae, both occurring in 14 patients. In nine cases, it was not possible to determine the cause of the bronchiectasis, and those cases were classified as idiopathic. In one patient each, there was a history of primary ciliary dyskinesia, Kartagener syndrome, cystic fibrosis, IgM deficiency, atypical mycobacteriosis, and post-bullectomy bronchiectasis in the setting of chronic obstructive pulmonary disease.

There was a symmetrical distribution among the lung lobes of the alterations reported by the two observers. Of the findings reported by observer 1, 18% were in the middle or right upper lobes, 20% were in the lower right lobe, 12% were in the left upper lobe, 15% were in the lingula, and 17% were in the left lower lobe, comparable to the 17%, 19%, 19%, 10%, 18%, and 17%, respectively, for observer 2 (Table 1).

**Table 1:** Results of agreement for each item evaluated.

HRCT findings	Interobserver agreement	Intraobserver agreement
Bronchiectasis		
Maximum size	κ = 0.45	κ = 0.54
Mean size	κ = 0.48	κ = 0.5
Appearance	κ = 0.43	κ = 0.47
Central distribution	κ = 0.48	κ = 0.46
Peripheral distribution	71.70%	κ = 0.51
Mucous plugging		
Central distribution	68.30%	κ = 0.39
Peripheral distribution	κ = 0.34	κ = 0.35
Bronchial wall thickening		
Severity	κ = 0.21	κ = 0.3
Central distribution	κ = 0.16	κ = 0.29
Peripheral distribution	61.30%	κ = 0.29
Involvement of the pulmonary parenchyma		
Opacity	κ = 0.39	71.90%
Ground glass attenuation	64.30%	κ = 0.24
Cists/bullae	κ = 0.47	κ = 0.44
Air trapping	47.80%	53.10%
Final score		
Intraclass correlation coefficient	71.90%	κ = 0.24
95% confidence interval	κ = 0.44	53.10%

**Figure 1:**
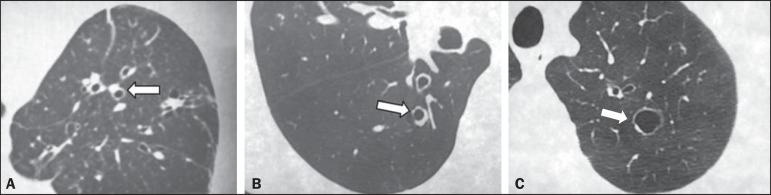
Chest HRCT scans of patients with cylindrical bronchiectasis (arrows). **A:** Bronchiectasis in the left lung, with an estimated bronchial caliber twice that of the adjacent artery. **B:** Bronchiectasis in the right lower lobe, with an estimated bronchial caliber three times greater than normal. **C:** Bronchiectasis in the upper left lobe, the largest with an estimated bronchial caliber four times greater than normal.

In the evaluation of the maximum size and mean size of the bronchiectasis (Figure 1), interobserver agreement was moderate, with κ values of 0.45 (*p* < 0.001) and 0.48 (*p* < 0.001), respectively. The intraobserver agreement was also moderate, with κ values of 0.54 (*p* < 0.001) and 0.50 (*p* < 0.001), respectively.

**Figure 2:**
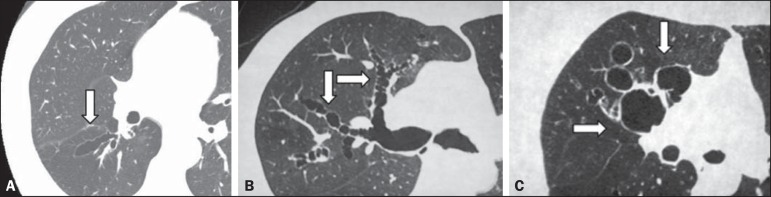
CT of the chest in patients with different patterns of bronchiectasis (arrows). **A:** Cylindrical central bronchiectasis. **B:** Varicose central bronchiectasis. **C:** Saccular central bronchiectasis.

In the classification of the appearance of bronchiectasis (cylindrical, saccular, or varicose) (Figure 2), there was also moderate agreement: κ = 0.43 (*p* < 0.001) for interobserver agreement; and κ = 0.47 (*p* < 0.001) for intraobserver agreement. For central distribution, the interobserver agreement was moderate (κ = 0.48; *p* < 0.001), as was the intraobserver agreement (κ = 0.46; *p* < 0.001). For peripheral distribution, the general interobserver agreement was 71.7% and the intraobserver agreement was moderate (κ = 0.51; *p* < 0.001). Central bronchiectasis was found by observer 1 in 64% of the cases and by observer 2 in 66%. However, peripheral bronchiectasis was found in 36% of the cases by observer 1 and in 34% by observer 2.

For the presence of mucous plugging, agreement was better when the location was central, with a general interobserver agreement of 68.3% and fair intraobserver agreement (κ = 0.39; *p* < 0.001) in the evaluation. For peripheral mucous plugging, interobserver agreement and intraobserver agreement were both fair—κ = 0.34 (*p* < 0.001) and κ = 0.35 (*p* < 0.001), respectively. Central mucous plugging was found in 42% of the cases by observer 1 and in 46% by observer 2, whereas peripheral mucous plugging was found in 58% of the cases by observer 1 and in 54% by observer 2.

The levels of interobserver and intraobserver agreement were lowest for the finding of severe bronchial wall thickening (κ = 0.21 and κ = 0.30, respectively; *p* < 0.001), especially when the thickening was centralized (κ = 0.16 and κ = 0.29, respectively; *p* < 0.001). In the analysis of peripherally distributed bronchial wall thickening, the interobserver agreement was better, with a general agreement of 61.3% and fair intraobserver agreement (κ = 0.29; *p* < 0.001). Central bronchial wall thickening was found in 78% of the cases by observer 1 and in 71% by observer 2. In contrast, peripheral bronchial wall thickening was found in 22% of the cases by observer 1 and in 29% by observer 2.

For the findings of opacity in the pulmonary parenchyma, ground-glass attenuation, and cysts/bullae, the interobserver agreement was fair (κ = 0.39; *p* < 0.001), good (general agreement of 64.3%), and moderate (κ = 0.47; *p* < 0.001), respectively, comparable to the intraobserver agreement, which was good (general agreement of 71.9%), fair (κ = 0.24; *p* < 0.001), and moderate (κ = 0.44; *p* < 0.001), respectively.

For the patients in whom good-quality expiratory phase images were available (*n* = 10), the general interobserver agreement for the finding of air trapping was good (general agreement of 47.8%), although the intraobserver agreement for that finding was slightly better (general agreement of 53.1%).

**Figure 3:**
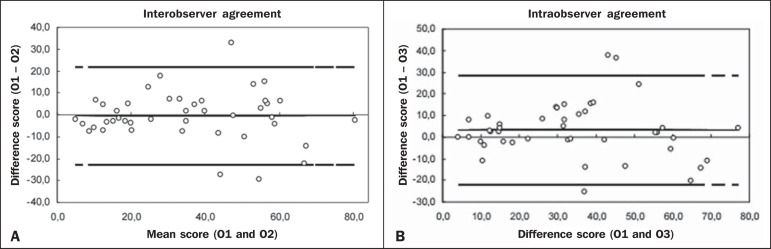
Interobserver agreement (**A**) and intraobserver agreement (**B**) for the final bronchiectasis score of each patient. The degree of agreement by the intraclass correlation coefficient can be considered excellent and is confirmed by the Bland-Altman graph, in which only a few points were outside the upper and lower limits established.

For interobserver agreement, the interclass correlation coefficient was 0.85 (95% CI: 0.74-0.91), compared with 0.81 (95% CI: 0.68-0.89) for intraobserver agreement (Figure 3.

## DISCUSSION

The use of HRCT scans to evaluate individuals with lung diseases has been the reason for a series of recent publications in the radiology literature of Brazil^(24-32)^. The results of the present study demonstrate that, in the HRCT evaluation of patients with bronchiectasis, interobserver and intraobserver agreement was generally moderate for each tomography finding in isolation. However, in obtaining the total score for each patient, the degree of agreement and reproducibility between the two evaluations was excellent.

Cylindrical bronchiectasis was the most common morphological pattern observed in the present study, a finding that is in agreement with data in the literature^(33)^. Varicose bronchiectasis can be confused with other morphological patterns if it is analyzed in a cross-sectional plane, which can mimic cylindrical and cystic bronchiectasis. These artifacts can explain the moderate interobserver and intraobserver agreement (κ = 0.43 and κ = 0.47, respectively) in the characterization of the morphological type of bronchiectasis.

Interobserver agreement and intraobserver agreement were both moderate for the detection of central mucous plugging, whereas they were both only fair for the detection of peripheral mucous plugging. This was probably due to the presence of other findings that could mimic the presence of "tree-in-bud" nodules, such as centrilobular nodules classified as areas of ground-glass attenuation or movement artifacts.

The thickening of the bronchial wall is a common finding in patients with bronchiectasis. One recent longitudinal study, in an adult population, showed that the severity of bronchial wall thickening was the main determinant of functional decline, implying that HRCT scans could be useful in monitoring the progress of the disease^(34)^. Numerous authors have proposed methods of qualitatively evaluating bronchial wall thickening^(35)^. However, none of those methods have gained widespread acceptance, and the definition of what constitutes abnormal thickening of the bronchial wall remains uncertain^(36)^. With the advent of multi-detector computed tomography (MDCT) devices, it became possible to identify bronchial wall thickening through the use of dedicated software^(37)^. In the present study, bronchial wall thickening was the finding for which the level of interobserver and intraobserver agreement was the lowest, principally when the thickening was centralized.

Some studies have shown that the interobserver agreement for the evaluation of bronchial wall thickening is worse than is that for the evaluation of bronchial dilation. Roberts et al.^(38)^ demonstrated near perfect interobserver agreement for the severity and extent of bronchiectasis (κ = 0.87 and κ = 0.82, respectively), and moderate interobserver agreement for bronchial wall thickening. Diederich et al.^(26)^ found poorer interobserver agreement for those same three parameters (κ = 0.78, κ = 0.76, and κ = 0.64, respectively).

In the present study, we found moderate interobserver and intraobserver agreement for the presence of pulmonary opacities, ground-glass attenuation, and cysts/bullae. The agreement for such findings could be better if the observers were more experienced in chest radiology, allowing greater distinction of the lesion patterns in the HRCT scans. However, we sought to reproduce the use of the quantification model for bronchiectasis by general radiologists, not thoracic imaging specialists, mirroring what would more commonly take place in daily practice.

In a study involving 70 individuals with bronchiectasis diagnosed by HRCT, air trapping was observed in 34% of the images obtained during expiration^(39)^. In the present study, the sample of patient in whom appropriate expiratory HRCT scans were available was small (*n* = 10), due to the technical difficulties for the acquisition of examinations of good quality. We found moderate interobserver and intraobserver agreement for the determination of air trapping, reflecting what is found in the literature^(35)^.

Some studies have demonstrated that MDCT, especially when performed with a 16-slice scanner, is superior to HRCT in the evaluation of bronchiectasis^(2,40,41)^. However, HRCT still plays an important role in Brazil, because it is more accessible. HRCT can be carried out on helical tomography devices, with a lower level of technological advancement than MDCT, without diminishing quality. Another advantage of HRCT scans is the acquisition of the image during a short breath-hold (with a duration of approximately 1 s) per tomography slice, whereas the acquisition of the fine MDCT slices of the whole chest requires a breath-hold of 10-15 s^(41)^. For dyspneic patients, this difference can be decisive in the quality of the image. In addition, there is a difference between the two methods in terms of the radiation dose, which is much higher in MDCT^(42,43)^.

As limitations of our study, we can cite the evaluation of images on tomography films, which limited the evaluation of the findings. The analysis of the examinations on a workstation would facilitate the differentiation between bronchiectasis and cystic lesions, for example, increasing interobserver agreement. However, there was no significant disadvantage for the final result by score.

In conclusion, we observed only moderate interobserver and intraobserver agreement in the evaluation of the tomography findings of bronchiectasis, when evaluated in isolation. However, in the final analysis of the findings, there were no significant changes in the classification of the severity of bronchiectasis for each patient. This shows the good reproducibility of the method for the quantification of bronchiectasis, with the aim of determining the severity of the disease.

The results of the present study, demonstrating an excellent correlation for the tomography score, according to the quantification model proposed by Brody et al.^(18)^, illuminate the possibility of correlating the quantification results and the tomography score with the clinical and functional data of the patients. This allows the HRCT of the chest to be considered a prognostic factor and serve as a tool for the non-invasive monitoring of adult patients with bronchiectasis.
